# Design and First Impressions of a Small Private Online Course in Clinical Workplace Learning: Questionnaire and Interview Study

**DOI:** 10.2196/29624

**Published:** 2022-04-07

**Authors:** Esther C Hamoen, Peter G M De Jong, Floris M Van Blankenstein, Marlies E J Reinders

**Affiliations:** 1 Department of Internal Medicine Leiden University Medical Center Leiden Netherlands; 2 Center for Innovation in Medical Education Leiden University Medical Center Leiden Netherlands; 3 Nephrology and Transplantation, Internal Medicine Erasmus Medical Center Transplantation Institute Erasmus Medical Center Rotterdam Netherlands

**Keywords:** blended learning, design-based research, web-based learning, workplace learning, medical education, clinical internship

## Abstract

**Background:**

Clinical workplace learning takes place in a dynamic and complex learning environment that is designated as a site for patient care and education. Challenges in clinical training can be overcome by implementing blended learning, as it offers flexible learning programs suitable for student-centered learning, web-based collaboration, and peer learning.

**Objective:**

The aim of this study is to evaluate the Small Private Online Course (SPOC) by interns’ first impressions and satisfaction measures (N=20) on using the SPOC. This study describes the design process of a SPOC from a theoretical and practical perspective and how it has been integrated into a clinical internship in internal medicine.

**Methods:**

The design of the SPOC was based on general theoretical principles that learning should be constructive, contextual, collaborative, and self-regulated, and the self-determination theory to stimulate intrinsic motivation. Interns’ impressions and level of satisfaction were evaluated with a web-based questionnaire and group interview.

**Results:**

Interns thought the web-based learning environment to be a useful and accessible alternative to improve knowledge and skills. Peer learning and web-based collaboration through peer interaction was perceived as less effective, as student feedback was felt inferior to teacher feedback. The interns would prefer more flexibility within the course, which could improve self-regulated learning and autonomy.

**Conclusions:**

The evaluation shows that the SPOC is a useful and accessible addition to the clinical learning environment, providing an alternative opportunity to improve knowledge and skills. Further research is needed to improve web-based collaboration and interaction in our course.

## Introduction

### Blended Clinical Workplace Learning

Clinical workplace learning (WPL) mostly takes place during normal daily collaboration and patient care activities, or organized formal learning activities [1,2]. It happens in a complex learning environment that is known to face many challenges. Patient cases tend to increase in complexity, whereas educational exposure is often insufficient, and time pressure leads to insufficient observation and assessment of the learner and suboptimal support within the diagnostic process [3-6]. Another challenge is the lack of sustained relationships among students, teachers, and patients [7,8]. These challenges, among others, lead to suboptimal clinical training.

Blended learning can be used to remedy several of these problems. Blended learning refers to a deliberate blending of face-to-face and web-based learning, with the goal of stimulating and supporting learning [9]. When thoughtfully designed, blended learning can improve education [10]. It may shift education to a more active and learner-centered approach, where the learner is *in control*, and may better fit the needs of different learning styles that students might have [11-13]. Overall, blended learning is more effective than traditional learning and, when carefully designed, has been demonstrated to have better effects on knowledge outcomes, learner motivation, and satisfaction than traditional face-to-face learning [10,14-17].

The web-based component of blended learning permits flexible education at a time, place, and pace convenient for the learner [18]. It can also help learners to share knowledge and experiences through web-based discussion forums and collaborative assignments with others although geographically dispersed. As web-based learning is complementary to instructor-led training, it can best be integrated in a blended learning curriculum [18,19]. The web-based component of blended learning in medical education can help students develop clinical reasoning skills by adding web-based patient experiences to real-world patient exposure.

### Small Private Online Courses

A Small Private Online Course (SPOC) is one possible instrument to blend web-based learning with clinical WPL. The SPOC concept was first introduced in 2013, and it has been progressively implemented in higher education thereafter. This type of course is often used locally with on-campus students and has a limited number of students that can enroll in the course [20]. Previous reports have shown that SPOCs can be feasible and suitable environments for student learning and fulfill students’ need for social interaction [19,21-23]. In medical education, SPOCs can positively impact professional practice and are thought to improve the management of patients [21,24]. It was shown that SPOCs need a flexible program and supportive environment to make them work [18]. SPOCs are relatively new in clinical WPL, and much is still unknown about how SPOCs can be optimally developed and integrated in clinical WPL. This information is required to improve the deployment of such blended programs in clinical training and in the end to improve the training of our future physicians.

The development of a dedicated SPOC instead of using publicly available course materials has the advantage that several secondary conditions such as contents definition, availability to interns, the alignment of goals, desired teaching modes, and assessments can be addressed by design. This avoids many of the current challenges with using open web-based education from others as described by de Jong et al [25] and Hendriks et al [26,27], such as limited constructive learning and a lack of certain desired teaching modes.

### Background and Objective

In 2017, we developed a SPOC for the Internal Medicine internship at Leiden University Medical Center (LUMC) in the Netherlands. The theoretical framework of the course is based on the self-determination theory (SDT) [28] and the general learning principles that learning should be constructive, contextual, collaborative, and self-regulated [29]. SDT offers a framework for driving intrinsic student motivation by stimulating autonomy, competence, and relatedness [28,30]. In the SPOC, groups of interns work on authentic clinical scenarios, use resources, and discuss with peers and teachers on a forum. The SPOC has been fully integrated into WPL; this means that knowledge and skills that are trained on the web can be directly transferred to the clinical environment where the interns have practical training and vice versa. In this paper, we evaluate the final design of the SPOC from a theoretical perspective. We report on the perceptions of interns on using this web-based resource in clinical WPL and their level of satisfaction. With the results, we hope to gain insight in the added value of introducing the SPOC in the WPL environment of our clinical internship.

## Methods

### Context

In the Netherlands, medicine students enter medical school at the bachelor level, which is followed by internships at the master level. At LUMC, each month approximately 20 interns start their clinical internships. In the first 4 weeks, the interns attend a joint program at the university. The so-called introductory internship (2 weeks) prepares them for the internships in general and is being followed by a specific 2-week theoretical course as preparation for the Internal Medicine internship. Thereafter, they start their clinical internship in Internal Medicine (12 weeks), in which they work in different affiliated hospitals in the region and cannot meet each other physically. A major aim during the internship is to obtain clinical reasoning skills for a broad range of clinical scenarios. However, the interns only have limited exposure to new patients who have not yet been diagnosed by other physicians, and the clinical scenarios that interns face in practice do not cover all the clinical scenarios that they need to know and understand. Collaboration between peers is limited because the group of interns is split up to have their internship in different hospitals.

### Design of the SPOC

#### Overview

To overcome several of the limitations mentioned, a SPOC has been developed. The course was designed using a design-based research approach in which practical and theoretical aspects are integrated in the educational design [29,31]. Attending the SPOC is facultative but highly recommended. However, once an intern decides to participate, several of the learning activities are obligatory to complete the lessons.

#### Practical Aspects

For the development of the course, a group of stakeholders has been identified including clinical interns, clinical teachers, educational experts, technical experts, and a graphic designer. During a dedicated learning experience design (LED) session the stakeholders set a framework for the SPOC, including the team and course’s goals (eg, improve patient expose), students’ needs (eg, track progression within the course), learning goals (eg, improve clinical reasoning skills), and the aimed look and feel (eg, authenticity of the cases).

#### Description of the SPOC

**Figure 1:**
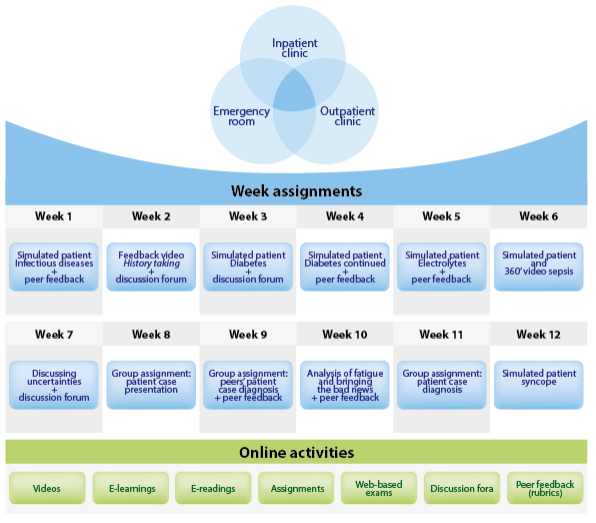
Small Private Online Course (SPOC) layout. Assessments during the 12 weeks of the clinical internship are displayed only (lessons presented in preparting for the internships and internal medicine are not displayed). The lessons include several inpatient, outpatient, and emergency room cases and assignments related to those participants.

**Figure 2:**
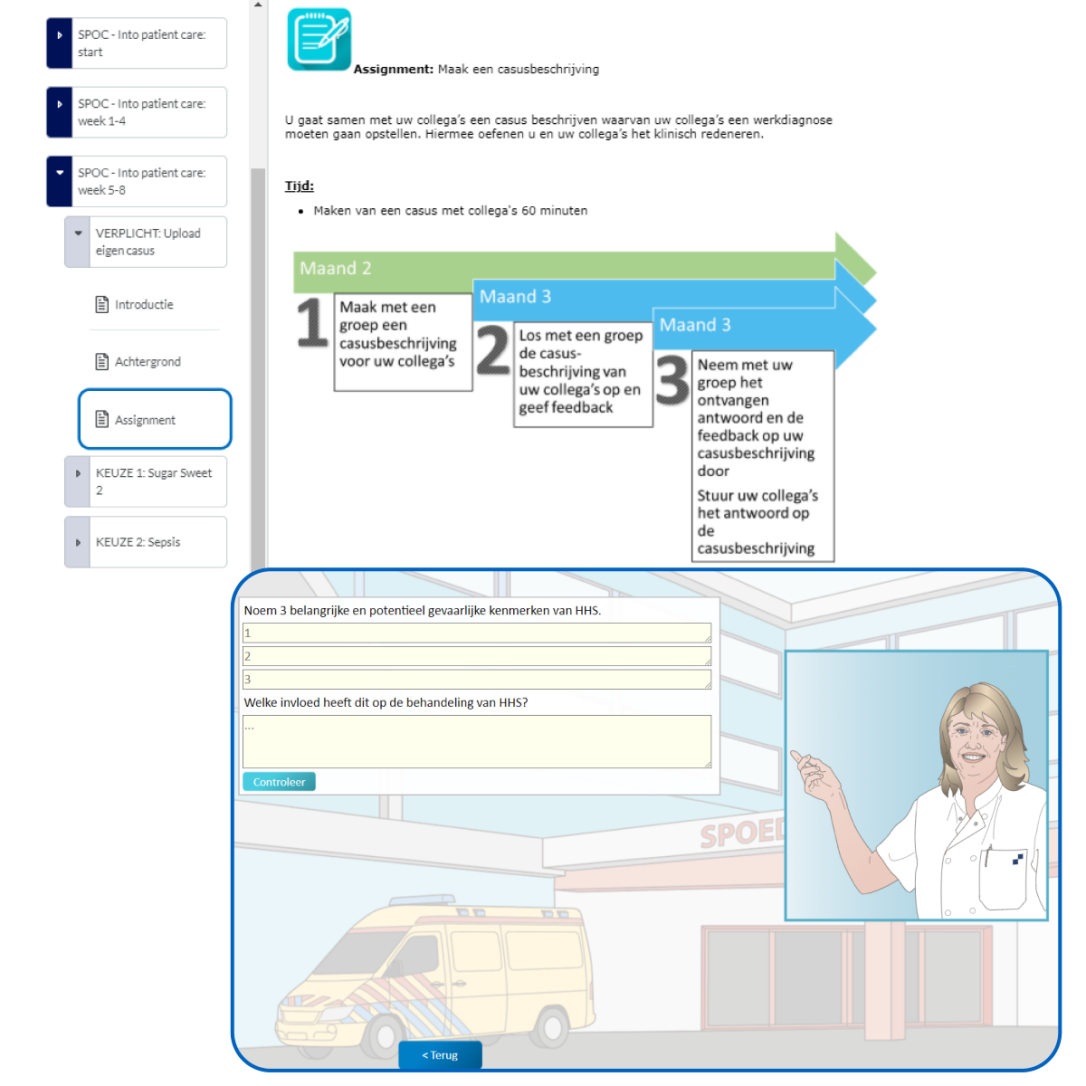
Exemplary screenshots of exercises in the Small Private Online Courses (SPOCs) as they are offered to the students.

The outcomes of the LED session were used to define the exact content and learning activities centered around authentic clinical problems that are typically encountered in internal medicine. NEO Learning Management System was used as a platform for the SPOC. Authentic cases in Dutch were developed to train clinical reasoning skills and medical knowledge. The SPOC has a modular design that involves preparing for the internships (2 weeks), internal medicine (2 weeks), and several inpatient, outpatient, and emergency room cases (12 weeks). Every week, the interns can study 1 new activity. A course overview of the 12 clinical weeks is shown in Figure 1. The lessons consist of various obligatory and optional assignments and learning activities including simulated patient cases, virtual reality applications (a virtual reality ward experience and professional or unprofessional behavior experience), group assignments, videos, e-learnings, e-readings, assignments, web-based exams, discussion forums, and peer feedback sessions. Exemplary screenshots of the intervention can be found in Figure 2. New knowledge can be directly applied to the clinical workplace where the interns work. Interns’ progression is tracked in the course, and it offers access to resources they can use in the workplace.

#### Theoretical Aspects

On the basis of the SDT and the general learning principles that learning should be constructive, contextual, collaborative, and self-regulated, authentic patient cases that are centered around virtual reality patients with complaints of diabetes, electrolyte disorders, infectious diseases, oncological diseases, and tiredness in a setting of the inpatient clinic, outpatient clinic, or emergency room have been developed. A 3D virtual reality patient ward has been developed, giving the interns a realistic impression of the inpatient setting. It aims to introduce interns to the ward and ward rounds and train professional standards such as hygiene regulations. Constructive learning activities such as doing rounds on 3D virtual reality patients with increasing complexity are used to gather knowledge and to improve learning effectiveness in the workplace.

Relatedness is promoted in the SPOC, as the interns (who work in different hospitals and do not have contact) meet up on the web on discussion forums, during peer feedback sessions and group assignments. This stimulates collaboration and relatedness among interns who would normally not meet each other during the internship. Collaborative forum assignments are included for peer learning and direct feedback by both peers and dedicated clinical teachers. Peer feedback is either given through open peer discussion on a forum or using a rubric format. Peer discussion, in this case on the web, might help interns to develop their critical thinking and clinical reasoning skills. Interns are prepared on their role as assessors by a web-based peer feedback training.

Besides obligatory content, the SPOC contains several optional assignments and resources that interns can choose from. The roster during the internship is different for each intern. An intern can spend 2 weeks in the cardiology department, whereas another intern works in the emergency room. Therefore, the interns can choose several sequential assignments for more extensive learning, depending on the training they need at that specific moment. By stimulating autonomy and attention to precourse goal setting and tracking and ranking course activity, it is aimed that interns can control their own learning process.

**Figure 3:**
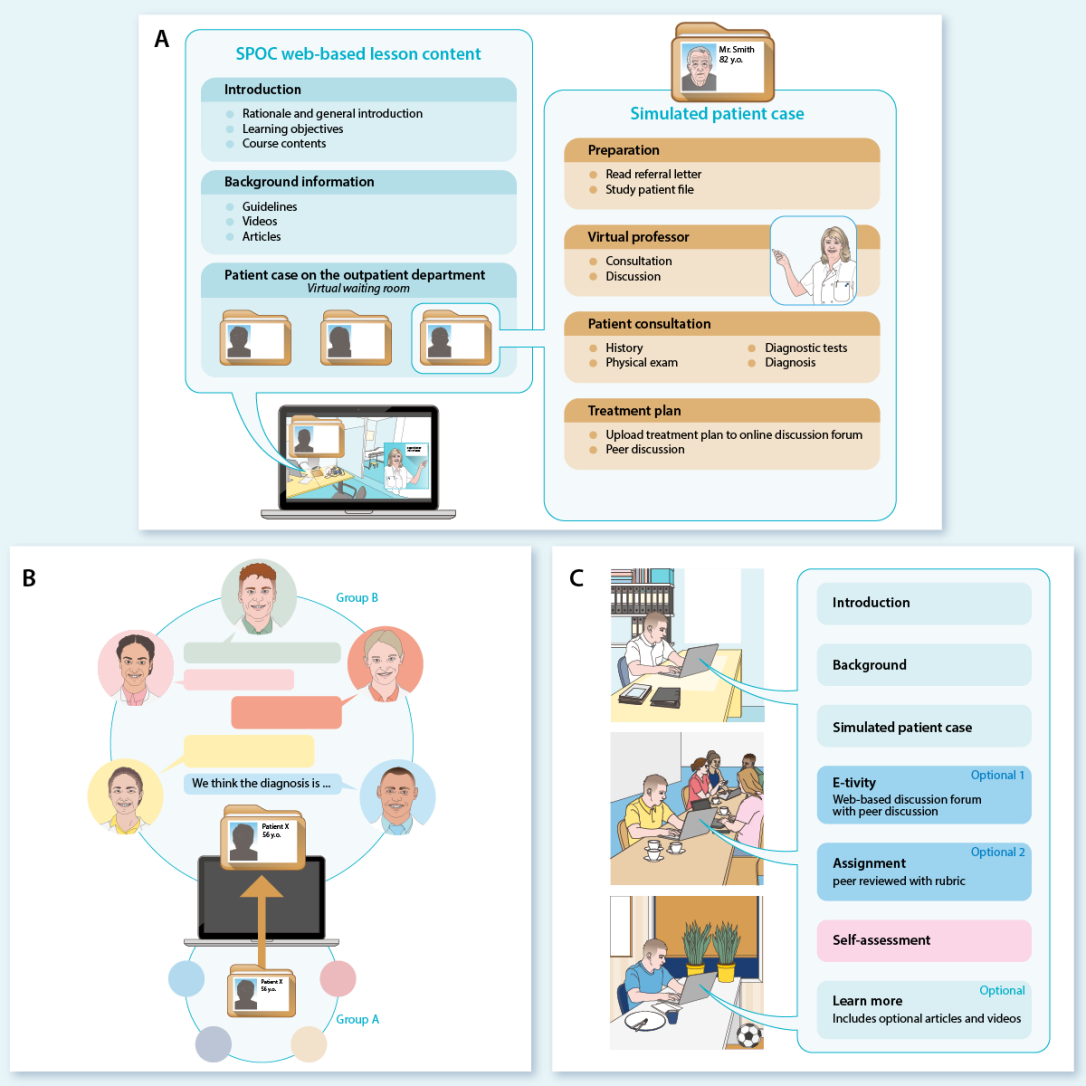
Examples of theoretical integration in the Small Private Online Course (SPOC). (A) Constructive learning in the clinical context. The figure illustrates how interns go through an authentic simulated patient case in the SPOC. The learning objectives are defined for each learning activity separately. For example, in one of the diabetes cases, the learning objectives are defined as follows. By the end of this lesson, the intern (1) is able to distinguish between type 1 and type 2 diabetes based on epidemiology, history, physical examination, and additional tests; (2) knows the complications of diabetes mellitus and knows the screening protocols; (3) knows the general treatment modalities for diabetes mellitus and cardiovascular risk management; and (4) knows the chain of care for patients with diabetes and knows the physician’s role within the chain. (B) Collaborative learning and relatedness. This figure displays a group assignment in the SPOC. A clinical scenario is described by one group of interns (group A), and another group of interns (group B) elaborates their clinical reasoning process that is completed by a diagnosis. Thereafter, feedback is provided by both groups on the quality of the case and the diagnosis, respectively. (C) Self-regulated learning or autonomy. The courses’ lessons contain required and facultative issues for further learning.

Training and assessment of competencies occur through peer feedback and self-assessment. Interns that have self-assessed their competencies and know that they are on the right track feel more confident while carrying out their new skills on real patients. In this blended program, skills that are trained on the web can be directly transferred to clinical practice. Practical examples of the integration of theory in the SPOC are shown in Figure 3.

### Participants

Participants were a first group of 20 interns who worked with the SPOC during a pilot period. They enrolled during their introductory internship and remained in the SPOC until completion of their Internal Medicine internship. The participants were in the first year of their clinical phase. In this phase, interns are aged approximately 21 to 25 years, and, on average, 60% to 70% are women. As the aim is to evaluate the impressions of the first group, no sample size calculation has been performed.

### Instruments

The evaluation existed of a web-based questionnaire (Multimedia Appendix 1) and an interview. The questionnaire consisted of 15 Likert-scale questions and 5 open-ended questions. The questionnaire contained questions that were mostly based investigation of the SPOC’s design; for example, what are the interns’ impressions of the aspects of the LED session, the SDT, and learning principles that were used in the SPOC’s design? It contained questions about the amount of time invested in the SPOC and whether the interns thought the SPOC to be useful, informative, and motivating. Interns were also asked whether they experienced more patient exposure while working in the SPOC and if they thought the cases were authentic. The questionnaire also contained questions about perceived competence, relatedness, and autonomy in the course. The educational context was quite unique, and therefore, there was no existing validated instrument available to measure the intended outcomes.

### Ethics Approval

As the study did not involve patients and no health intervention has been administered to participants, the study was not subjected to the Dutch WMO (Medical Research involving Human Subjects Act). The study has been conducted in compliance with the European Union General Data Protection Regulation 2016/679, and data have been anonymized and stored according to the *Nederlandse Gedragscode Wetenschapsbeoefening* of the Universiteiten van Nederland (Association of Universities The Netherlands). Institutional educational review board approval was obtained under reference OEC/ERRB/20220208/1. All interns provided written informed consent to participate in the study. In the information letter, the full study procedure was explained, as well as the option for the interns to opt out of the study at any moment without any reason.

### Procedure

The students completed the weekly assignments and remained in the SPOC until they had completed their Internal Medicine internship. Subsequently, they were asked to fill out the web-based questionnaire. A group interview was led by an independent interviewer who had no intern-teaching relationship with the interns. The interview used the *snowball method*: the participants first individually recalled their own experiences with the course, and then 2 participants paired up to discuss their experiences and wrote down their most important findings, which was repeated in groups of 4 participants. All the experiences were shared with the group by oral presentation. The interviews were audio recorded and converted into a transcript by the first author (ECH).

### Data Analysis

#### Questionnaire

Means and SDs were calculated for the Likert-scale questions in the web-based questionnaire. The answers to the 5 open-ended questions were summarized.

#### Interview

The first and third authors discussed the transcript coding template, which was based on the different items in the theoretical framework, until consensus was obtained. The outcome was a template consisting of six predefined, overarching themes: (1) contextual learning, (2) collaboration and relatedness, (3) constructive learning, (4) self-regulated learning or autonomy, (5) competence, and (6) other. For each category, the same authors agreed on a definition for each theme. The principal investigator (ECH) analyzed the interview data and clustered the answers in the template using a Microsoft Word.

## Results

### Collected Data

The aim of this study is to investigate the perceptions of interns concerning the use of the SPOC using a questionnaire and interview. Of the 20 interns eligible to enroll in the SPOC, 19 (95%) actually enrolled in the course, 10 (50%) finished the whole course, and 1 (5%) never started. Ten interns filled out the web-based questionnaire. Only questionnaires that were fully completed were included in the analysis. All 20 interns attended the group interview.

### Questionnaire: Likert-Scale Questions

The results are shown in Table 1. The SPOC was valued as being informative and useful to most of the interns, and they felt that the patient cases were authentic. However, the interaction with peers was found inadequate and not useful. Interns’ perceptions on motivation to learn in the SPOC was not optimal.

**Table 1:** Outcomes of the web-based questionnaire (n=10).

Question	Value, mean (SD)^a^
1. Time per week spent in the course (hours)	1.8 (0.92)^b^
2. Following the course is useful during the internship	4.5 (1.96)
3. The course was informative	5 (1.33)
4. The SPOC^c^ was a motivation for learning	3 (1.49)
5. I would recommend the course to peer students	3.9 (1.91)
6. The knowledge obtained from the SPOC was fairly applicable to clinical practice	4 (1.89)
7. Through working in the SPOC, my patient exposure has been increased	3.3 (1.64)
8. The cases in the SPOC were authentic	4.8 (1.23)
9. Making the cases, I really felt like a physician making decisions	3.6 (2.01)
10. I felt more competent in clinical reasoning after finalization of the SPOC	4.1 (1.79)
11. The SPOC had a good construction of increasing difficulty	4.7 (1.06)
12. I could organize my own time well within the SPOC	4.1 (1.79)
13. In the course, I had good interaction with peers	2.4 (1.71)
14. The interaction with peers was useful	1.8 (1.03)
15. The assignments and tests were challenging	4.3 (1.70)

^a^The 7-point scale ranges as follows: (1) totally disagree to (4) neither disagree nor agree to (7) totally agree.

^b^Note that the number of of hours spent per week is displayed in this row.

^c^SPOC: Small Private Online Course.

### Questionnaire: Open-ended Questions

The interns highlighted the patient cases as a positive aspect of the course. They particularly valued their connection with clinical practice and the elaboration and variety of relevant cases. Furthermore, the interns appreciated the group assignment in which they *solve* a patient case by finding the diagnosis. Interns liked the graphical layout of the course. The SPOC supported training clinical reasoning skills, although some interns felt that those skills are better trained in practice or when the specific SPOC cases were also experienced in clinical practice. Concerning peer feedback, the interaction during the patient cases was thought to be the most useful, as were the feedback training and having insight in the answers of peers. Some interns indicated that the technology to give feedback did not always work well or that interaction in clinical practice was more useful. The information about deadlines, the instruction for how to give peer feedback, the quality of the feedback received from peers, and the fixed order of the assignments were mentioned as limitations of the course. Owing to time constraints, some interns indicated they had difficulties finalizing the mandatory contents within the lessons. They would have preferred optional content only.

### Interview

The interns were interviewed in their last week of the internship. Table 2 shows a more detailed overview of the results of the group interview. Constructive and collaborative learning were clustered under 1 category because the interview data overlapped in both categories.

The interns experienced the SPOC to be accessible and adequately designed for them as a target group. The participants felt the patient cases and SPOC content were useful and informative. However, although authenticity was integrated in the design of the SPOC, the participants felt that the contents did not match the real world. It seemed unclear to them how the SPOC should complement clinical WPL. They would prefer assignments that matched the clinical problems they encountered at that time instead of fixed weekly assignments.

Development of critical thinking skills by peer discussion was also integrated in the SPOC’s design; however, the interns indicated that they preferred model answers over peer discussion. The participants also felt it was not really useful to receive feedback from peers instead of a teacher, because in their opinion peers know as much as they do themselves. In general, feedback was perceived to be very short.

The interns indicated they had enough time to finish the assignments during their normal day shifts, although not everyone agreed. They also appreciated the facultative character of the SPOC. They felt that more facultative assignments would be helpful, although they addressed the possibility that nobody would mind finishing them. They also indicated that they needed more flexibility and choice in the course, and the fixed order of the assignments did not work for everyone. Some participants felt they had “finally reached the clinical phase of their internship” and therefore did not appreciate completing web-based assignments in this stage of the curriculum. They also preferred a complete overview of a certain clinical presentation instead of looking up information themselves through links in the SPOC.

**Table 2:** Group interview concerning SPOC^a^ perceptions^b^.

Theme	Positive	Negative
Contextual learning	SPOC was adequately targeting interns Useful patient cases Content was handy and informative	Assignments did not match clinical problems encountered in WPL^c^ Learning objectives were unclear Unclear how SPOC complements WPL Contents did not match the real world
Collaboration, relatedness, and constructive learning	—^d^	Peer feedback is less useful than feedback from teacher Interaction Interns wanted answer sheets instead of peer discussion
Self-regulated learning or autonomy	Sufficient time to finish assignments during daily shift Nice that SPOC is not obligatory	Tight deadlines Time-consuming Insufficient time to finish assignments during daily shift Need more choice instead of fixed assignments Do not want theoretical assignments during the practical phase Less obligatory and more optional assignments Want a complete overview of a clinical presentation, instead of looking up information Wished the SPOC to be more motivating
Competence	Good patient cases, e-learnings, and quizzes Useful lessons, mainly virtual reality patients Useful for interns with less patient contacts and less moments for clinical reasoning Enjoyed videos observing others taking history History taking videos led to discussion among peers Those videos must be part of the training	SPOC did not fill gaps encountered in clinical practice Watching videos seeing others taking history is not active learning Learned more from observation in workplace Need more specific physical exam tools instead of observation general physical examination Need more assignments that specifically enhance knowledge
Other	SPOC was accessible Good learning environment Videos were enjoyable	Unclear deadlines Problems planning patient-related assignments (suitable patients dismissed) Technical shortcomings Insufficient support

^a^SPOC: Small Private Online Course.

^b^The positive and negative perceptions of the interns are displayed in the middle and right columns. Those were assembled in 6 predefined clusters (left column). Collaboration and constructive learning have been clustered because of overlapping interview data.

^c^WPL: workplace learning.

^d^Not available.

The interns particularly valued the competence training received through completion of the patient cases, e-learning, and quizzes. The virtual reality patient cases and videos were rated as useful and likable. The videos concerning observation of patient interviews were enjoyable and led to useful discussion between peers. However, others felt that observation in clinical practice is more informative than watching web-based videos. Although the virtual reality patient cases can be an alternative for practicing clinical scenarios that have not been encountered in live patient contact, the interns indicated that the SPOC did not completely fill the gaps owing to the limited number of virtual reality patient cases addressed in the SPOC.

In general, the interns appreciated the accessibility of the SPOC and its learning environment, and the videos were enjoyable. They were less content about the deadlines that sometimes appeared to be unclear and the planning of SPOC activities that were directly patient related, which was challenging because of fast patient turnover rates. Completion of some of the assignments was difficult because of technical shortcomings or insufficient support.

## Discussion

### Principal Findings

In this study, a SPOC has been developed and implemented into an internship curriculum of Internal Medicine at LUMC. Course development was based on general learning principles and SDT, aiming to promote learner motivation and optimize the course’s quality and integration in the clinical curriculum. The SPOC was evaluated by measuring the satisfaction of the interns with the course. Interns thought the SPOC to be a useful and accessible addition to the clinical learning environment. Peer learning and web-based collaboration through peer interaction were perceived as less effective, as student feedback was felt inferior to teacher feedback. The interns preferred more flexibility within the course, which could improve self-regulated learning and autonomy. Overall, the interns felt that the web-based learning environment provided an alternative opportunity to improve knowledge and skills.

Our SPOC was integrated into clinical practice by making it part of the internship program, instead of using it as a stand-alone web-based training. The advantage of this is that learners can directly apply their new knowledge in clinical practice and go back to the web-based resources for further exploration of a topic. In our opinion, using design sessions and a theoretical framework can facilitate integration of such a course into an existing curriculum and can be an effective solution for some of the complexities faced in clinical training.

From the results, we learned that the SPOC meets the expectations for some of the categories fairly well, although the overall student satisfaction seems to be modest. In general, completing the SPOC assignments during the internships seemed feasible but not for all interns. Concerning learning in the clinical context, interns appreciated the authenticity and usefulness of patient cases as an additional opportunity to improve their clinical knowledge and skills. Regarding the clinical skills development, the SPOC therefore meets our hypothesis; however, this is not the case for stimulation of the social cohesion of the interns through working in the SPOC. The quantitative data show a modest to very low outcome on several topics (score ≤3.3: motivation, interaction, and usefulness of interaction with peers and feeling like a physician making decisions).

It appeared that the interns were not satisfied with the collaboration and relatedness aspects of the SPOC, in particular the web-based interaction and peer feedback. Other studies confirm that students prefer teacher feedback over peer feedback [32-34]. When peers lack knowledge or are not critical, the peer feedback is often considered inadequate [35]. In our SPOC, peer feedback was a prespecified learning objective and was supposed to be a formative process to promote learning. It is possible that the interns perceived peer feedback merely as a mandatory assignment for completing the SPOC and that they did not understand its importance in their own learning process and that of their peers. Other studies describe that guidance on assessment and the requirements hereof, training in giving peer feedback, and clarification of the role the student takes in the feedback process are key principles of effective feedback [36,37]. Furthermore, it is important to explain the purpose of peer feedback to the students [38]. Despite the fact that our SPOC contains a peer feedback training, we might need to strengthen the guidance of the interns in their role as peer assessors. In addition, the learning objectives of the peer-assessed activities could be clearer on the purpose providing and receiving feedback.

Learning activities in a SPOC are on the web and asynchronous, with only written interaction and no visual interaction and body language [39]. This may have affected the learning experience of the interns, as interaction on cognitive, social, and teaching levels is required to promote deep learning [40]. Possible interventions that might improve interaction in these three levels are more visual teacher presence in our SPOC, synchronized learning activities to stimulate just-in-time learning, more insight into interns’ learning needs, adaptivity of the teaching strategy, social cohesion, and promoting deep learning by considering feedback as a dialogical process while considering asynchronous learning in the SPOC [39,41].

Although flexibility was integrated in the design of the course based on previous literature [18], interns indicated they needed more flexibility and that deadlines were too tight. They also requested more autonomy in choosing when to complete which lessons. Self-regulated learning skills are essential in web-based learning owing to the freedom provided in web-based education. It gives learners autonomy in how they organize their learning, and they need to deal with that to be successful [42]. Our evaluation was conducted among undergraduate medical students. Previous studies have shown that self-regulation, such as experienced in e-learning, might not fit novice learners that lack the maturity and experience to reach learning outcomes that are minimally guided [43-45]. Novice students may lack the cognitive, affective, and metacognitive self-regulated learning skills necessary to effectively navigate the abundance of information that is nonlinearly provided by the hypermedia [46]. This may affect the learners’ acceptability and satisfaction of e-learning activities and consequently their emotional experience. Self-regulated learning within web-based learning environments is also influenced by the emotional experience of the students [47]. Studies found a negative relation between negative emotions and learning outcomes, and the emotional experience and subsequent learning may be improved by fostering students’ emotion regulation [47-49]. The technical shortcomings of the SPOC may have attributed to a negative emotional experience of our interns.

Regarding the feelings of being a physician making decisions and contextual learning, it seemed that the SPOC somehow failed in this aspect. In the LED session it was predefined to make authentic simulated patient cases to enhance this feeling and the feeling of increased patient exposure; however, this was not experienced by the interns. Although studies have shown that virtual reality patients can improve clinical reasoning skills and knowledge, it should be recognized that patient simulations are not equivalent to real patients and cannot replace traditional clinical WPL [50-52].

### Reflection and Improvements

Reflecting on our study, this may imply some improvements in our SPOC’s design. First of all, we should critically review the technical shortcomings, deadlines, and obligatory components of the SPOC and improve the flexibility of the learning environment. For instance, replacing the weekly assignments by a more variable set of assignments on a monthly basis could be considered. We are currently investigating the feasibility of such adaptations and the expansion of the number of optional lessons. In addition, the SPOC will soon be transferred to another internet platform, which is technically supported by our University’s Information Technology department.

Second, it may be helpful to incorporate training of self-regulated learning skills before using our hypermedia environment and more support and augmentation of learners’ self-regulated learning within the learning system [46,48,53]. From the interview data, we also learn that the SPOC’s general instructions were valued as suboptimal. Therefore, a stronger instructional guidance and guidance of learners’ self-regulated learning in our SPOC may improve the effectiveness of the learning experience and learner satisfaction [46,48,53,54]. In addition, incorporation of emotion regulation in the learning activities of such a SPOC and in the design and implementation in clinical WPL may improve self-regulated learning and maximize positive effects on students’ learning within the digital learning environment [47]. These topics might be subject to future research. We believe that, as discussed, more focus on self-regulated learning, the emotional experiences within the SPOC, guidance, and the purpose of feedback may also improve the students’ motivation for learning within the SPOC.

Third, several other conditions may be met to increase student satisfaction. Student satisfaction in clinical internships is enhanced by supervisor support, perceived social value [55], dedicated faculty, working in teams, and continuity in intern-patient contacts [56]. Longitudinal relationships between supervisors and students also increase student satisfaction [57,58] and students’ independence [59]. Therefore, for instance, dedicated supervisors could provide feedback in the SPOC in a longitudinal working relationship with their interns. In addition, students might be encouraged to use the learning resources in the SPOC in continued contacts with patients. This may promote the SPOC’s social value.

### Limitations

When critically looking at the quantitative data, it is apparent that the variance is modest and particularly low for the questions concerning the interaction with peers and increasing difficulty. An explanation might be that the interns know each other fairly well and their answers may not be independent or even biased by information, sharing leading to a group opinion. Limitations of the study were the small sample size and use of an unvalidated questionnaire. As the study was designed just to evaluate the first group of interns enrolled in the SPOC, no formal sample size calculation has been performed. However, we did include qualitative data to supplement these limited quantitative outcomes. We therefore think that the impressions still provide valuable insights in the SPOC’s strengths and weaknesses that we can use for further adaptation.

### Conclusions

From our study, we have learned that interns perceive several learning opportunities after adding the SPOC to their clinical learning environment, mainly in skills and knowledge acquisition. However, particularly web-based collaboration and perception of relatedness among the interns within the course need further improvement. In the future, interviews with the interns may be beneficial for a deeper investigation of this issue and others, their context, and which additional adaptations might be needed to our SPOC. Future research is also needed to further investigate how learning principles can be optimally integrated in web-based courses.

## Appendix

Multimedia Appendix 1 Questionnaire.

## Abbreviations

LED: learning experience design
LUMC: Leiden University Medical Center
SDT: self-determination theory
SPOC: Small Private Online Course
WPL: workplace learning
