# Supplementary material for: Design and First Impressions of a Small Private Online Course in Clinical Workplace Learning: Questionnaire and Interview Study
Source: JMIR Med Educ. 2022 Apr 7;8(2):e29624. doi: 10.2196/29624 (PMC9030912; doi:10.2196/29624)
Supplement: Multimedia Appendix 1 [file mededu_v8i2e29624_app1.docx]

# **Appendix I Questionnaire**

## **General**

1. How many hours of self-study did you spend on the online course? *Think carefully and try to estimate this as accurately as possible.*

For each of the following statements, please indicate how much you agree with this, using the following scale:

1. Following the course is useful during the internship.
2. The course was informative.
3. The SPOC was a motivation for learning.
4. I would recommend the course to peer students.

## **Clinical practice**

For each of the following statements, please indicate how much you agree with this, using the following scale:

1. The knowledge obtained from the SPOC was fairly applicable to clinical practice.
2. Through working in the SPOC my patient exposure has been increased.
3. The cases in the SPOC were authentic
4. Making the cases, I really felt like a physician making decisions
5. I felt more competent in clinical reasoning after finalization of the SPOC (use 7 pt scale). Motivate your answer

## **Learning principles**

For each of the following statements, please indicate how much you agree with this, using the following scale:

1. The SPOC had a good construction of increasing difficulty
2. I could organize my own time well within the SPOC
3. In the course I had good interaction with peers
4. The interaction with peers was useful
5. Which form(s) of interaction did you find most useful?
6. The assignments and tests were challenging

## **Final questions**

1. What did you think were the strengths of this online course?
2. Do you have any tips to improve this online course?
3. Do you have any other additional comments about this online course?
